# Abnormal diastolic function underlies the different beneficial effects of cardiac resynchronization therapy on ischemic and non-ischemic cardiomyopathy

**DOI:** 10.6061/clinics/2017(07)08

**Published:** 2017-07

**Authors:** Qi Wang, Kang-Yu Chen, Fei Yu, Hao Su, Chun-Sheng An, Yang Hu, Dong-Mei Yang, Jian Xu, Ji Yan

**Affiliations:** I Department of Cardiology, Provincial Hospital Affiliated to Anhui Medical University, Hefei, Anhui, China; II Cardiovascular Institute of Anhui, Hefei, China

**Keywords:** Heart Failure, Cardiac Resynchronization Therapy, Diastole

## Abstract

**OBJECTIVES:**

To investigate the association between diastolic function and the different beneficial effects of cardiac resynchronization therapy in patients with heart failure due to different causes.

**METHODS:**

The 104 enrolled patients were divided into an ischemic cardiomyopathy group (n=27) and a non-ischemic cardiomyopathy group (n=77) according to the cause of heart failure. Before implantation, left ventricular diastolic function was evaluated in all patients using echocardiography. After six months of follow-up, the beneficial effects of cardiac resynchronization therapy were evaluated using a combination of clinical symptoms and echocardiography parameters.

**RESULTS:**

The ischemic cardiomyopathy group included significantly more patients with restrictive filling than the non-ischemic cardiomyopathy group. The response rate after the implantation procedure was significantly higher in the non-ischemic cardiomyopathy group than in the ischemic cardiomyopathy group. Degrees of improvement in echocardiography parameters were significantly greater in the non-ischemic cardiomyopathy group than in the ischemic cardiomyopathy group. Multivariate regression analysis showed that a restrictive filling pattern was an independent factor that influenced responses to cardiac resynchronization therapy.

**CONCLUSIONS:**

This study again confirmed that the etiology of heart failure affects the beneficial effects of cardiac resynchronization therapy and a lower degree of improvement in ventricular systolic function and remodelling was observed in ischemic cardiomyopathy patients than in non-ischemic cardiomyopathy patients. In addition, systolic heart failure patients with severe diastolic dysfunction had poor responses to cardiac resynchronization therapy. Ischemic cardiomyopathy patients exhibited more severe diastolic dysfunction than non-ischemic cardiomyopathy patients, which may be a reason for the reduced beneficial effect of cardiac resynchronization therapy.

## INTRODUCTION

Cardiac resynchronization therapy (CRT) can improve symptoms, increase quality of life, reduce the risk of hospital readmission, and decrease the mortality of chronic heart failure patients 1. However, approximately 30% of patients present non-response to CRT based on current standard guidelines 2. Substantial evidence-based medical data indicate that the beneficial effect of CRT depends on the etiology of heart failure. The beneficial effects of CRT are smaller in patients with ischemic cardiomyopathy (ICM) than in patients with non-ischemic cardiomyopathy (NICM); therefore, ICM is a predictive factor for CRT non-response 3,4. Given this difference in the beneficial effects of CRT for different patients, prior studies have examined quantity of viable myocardium, myocardial scar burden, degree of scar transmurality, scar location, and left ventricular (LV) pacing to assess the potential relevance of these factors 5-7. However, the specific mechanism of these different effects remains unclear. Diastolic function is an important component of overall cardiac function 8. The process and degree of diastolic dysfunction differ in different etiologies. Based on previous findings, we hypothesized that ICM patients who meet CRT indications exhibit more serious diastolic dysfunction than NICM patients, which may be a reason for the differing beneficial effects of CRT.

## METHODS

### Study subjects

Consecutive patients who were scheduled to receive CRT pacemaker/defibrillator (CRT-P/D) treatment for chronic heart failure at Provincial Hospital Affiliated with Anhui Medical University from April 2013 to January 2015 were selected. Patients who met the following criteria were enrolled in the study 9: 1 grade III-IV on the New York Heart Association (NYHA) functional classification after treatment with standard drugs for heart failure; 2 sinus rhythm, left bundle branch block (LBBB), and QRS duration ≥120 ms; and 3 left ventricular ejection fraction (LVEF) ≤0.35. The exclusion criteria consisted of 1 atrial fibrillation; 2 acute myocardial infarction within three months before implantation; and 3 primary organic valvular heart disease. A total of 106 patients were enrolled. Because two patients suffered LV lead implantation failure, 104 patients were included in the statistical analysis. The NICM group consisted of 77 patients, and the ICM group comprised 27 patients. ICM was defined as ≥70% stenosis of at least one major coronary artery and its large branch on coronary angiography or a history of myocardial infarction.

### Clinical evaluation

The NYHA functional classification of patients was determined before implantation, and the disease course of heart failure, medications, and serum creatinine values were recorded. All patients underwent 12-lead electrocardiography (ECG) to determine heart rhythm, QRS duration, and morphology as well as echocardiography to evaluate cardiac structure and systolic and diastolic function. Six months after implantation, the NYHA functional classification was re-evaluated, and ECG and echocardiography were performed to assess the changes.

### Echocardiography

Each patient underwent conventional echocardiography before implantation and at six months after implantation. A Philips iE33 ultrasound system with an S5-1 probe was used (1.0-5.0 MHz).

Left atrial as well as LV end-systolic and end-diastolic diameters (LAD, LVESD and LVEDD, respectively) were obtained in M mode. LV volumes and the ejection fraction (LVESV, LVEDV and LVEF) were assessed using the biplane Simpson’s rule. The LV outflow tract-velocity time integral (LVOT-VTI) was measured within 0.5-1 cm below the aortic valve, and the systolic pulmonary artery pressure (SPAP) was estimated based on the tricuspid regurgitation velocity. LV dyssynchrony was assessed using the standard deviation of the time to peak systolic velocity in 12 LV segments (Ts-SD) derived from tissue Doppler images 10.

The degree of mitral regurgitation (MR) was evaluated according to the ratio between the maximal mitral regurgitant jet area and the left atrial area as follows: mild (Grade 1) <20%, moderate (Grade 2) 20-40%, and severe (Grade 3) >40%.

Diastolic function was assessed by transmitral Doppler and tissue Doppler. Pulse Doppler was used to acquire the diastolic mitral valve flow spectrum and record peak early diastolic velocity (E), peak late diastolic velocity (A), and diastolic early filling deceleration time (DT) and to calculate the E/A ratio. Tissue Doppler was used to acquire mitral annular velocity curves corresponding to the lateral wall and interventricular septum, to record mitral annular early diastolic peak velocity (Em lateral and Em septal), and to calculate the mean Em value and E/Em ratio. According to the mitral valve flow spectrum and the tissue Doppler spectrum of the mitral annulus, patients were divided into restrictive filling pattern (RFP) (E/A≥2, DT<160 ms, and E/Em≥13) and non-RFP (E/A<2, DT≥160 ms, and E/Em<13) groups 11.

### Placement and optimization of CRT

CRT was placed using the transvenous approach. Subclavian vein puncture was performed. After coronary sinus intubation, retrograde angiography was performed to fully display all branches of the coronary veins. The LV electrode lead was placed in the lateral or posterolateral cardiac vein to ensure pacing capture, with high voltage (10 V) used to demonstrate a lack of phrenic nerve stimulation. The right ventricular electrode lead was placed in the right ventricular apex or septum. The right atrial electrode lead was conventionally placed in the right atrial appendage. After the pacing tests were completed, the electrode leads were connected to a pulse generator and embedded in a subcutaneous or a deep to pectoralis major pouch in the chest wall. The incision was sutured in layers.

Within one week of implantation, atrioventricular (AV) delay and interventricular (VV) delay optimization were performed under echocardiographic guidance. AV delay optimization was conducted using the iterative method. In particular, the optimal AV delay was determined based on the maximum left ventricular filling times at 6 selected AV delays: 180, 160, 140, 120, 100, and 80 ms. VV delay optimization was performed after AV delay optimization; the optimal VV delay was determined using the maximum left ventricular outflow tract velocity time integral at peak velocity (LVOT-VTImax) 12.

Six months after implantation, a ≥15% reduction in LVESV compared with the preoperative value was defined as a CRT response.

### Statistical analysis

The statistical analysis was performed using SPSS 19.0 software. Continuous variables are presented as the mean±standard deviation. Categorical variables are presented as frequencies and percentages. Comparisons of data within groups were performed with paired Student's t-tests (continuous variables) and Wilcoxon signed-rank tests (NYHA classification, MR grade). Comparisons of data between groups were performed with unpaired Student's t-tests (continuous variables) and Mann-Whitney U tests (NYHA classification, MR grade). Comparisons of gender and CRT response rates between groups were performed with *x*^2^ tests. Influencing factors on CRT responses were analyzed using univariate and multivariate logistic regression models. A value of *p*<0.05 indicated a statistically significant difference.

## RESULTS

1. **Baseline information** (Table 1). In this study, 104 patients were enrolled, including 77 patients in the NICM group and 27 patients in the ICM group. Before implantation, age, gender, NYHA functional classification, PR interval, QRS duration, serum creatinine level, echocardiography parameters (LAD, LVEDV, LVESV, LVEF, SPAP, MR, LVOT-VTI, and Ts-SD), and drug treatment conditions were not significantly different between these two groups. Compared with the NICM group, the ICM group exhibited higher values of E/A and E/Em and shorter DT values (*p*<0.05 for all comparisons). The RFP was significantly higher in patients in the ICM group than in patients in the NICM group (51.9% *vs*. 24.7%, *x^2^*=6.816, *p*=0.009). One week after implantation, the AV delay optimization was not significantly different between the ICM group (119.3±19.6 ms) and the NICM group (123.1±17.2 ms).

2. **CRT response rate**. Six months after the implantation procedure, 66 out of the 104 patients (63.5%) exhibited responses to CRT. The CRT response rate was significantly higher in the NICM group than in the ICM group (70.1% *vs*. 44.4%, *x^2^*=5.688, *p*=0.017).

3. **Clinical beneficial effects of CRT**. NYHA functional classification, LAD, LVEDV, LVESV, LVEF, FS, and LVOT-VTI were improved six months after the implantation procedure relative to before implantation (*p*<0.001 for all comparisons). The degree of MR was lower than before implantation (*p*<0.05). The SPAP did not change significantly. A comparison of parameters before and after CRT is presented in Table 2.

In the NICM group, the postoperative NYHA functional classification, LAD, LVEDV, LVESV, LVEF, FS, and LVOT-VTI were all significantly improved compared with the values recorded before implantation (all *p*<0.001); the degrees of SPAP and MR were both lower than those recorded before implantation (both *p*<0.05). In the ICM group, the postoperative NYHA functional classification, LVESV, LVEF, FS, and LVOT-VTI were all significantly improved (all *p*<0.05), whereas the LAD, LVEDV, SPAP, and MR did not exhibit significant changes.

Six months after implantation, the differences in echocardiography parameters (LVESV, LVEF, FS, SPAP, and LVOT-VTI) between the two groups were significant (all *p*<0.05). A comparison of parameters between the NICM and ICM groups is shown in Table 3.

4. **Factors that influence the CRT response** (Table 4). The univariate logistic regression analysis results suggested that male gender, an etiology of ischemia, a disease course of heart failure, serum creatinine level, QRS duration, LAD, SPAP, severe MR, and RFP influenced the CRT response. When these factors were included in the multivariate logistic regression analysis, the results showed that QRS duration, LAD, and RFP were independent factors that influenced the CRT response.

## DISCUSSION

This study showed that 1 the improvement of left ventricular systolic function and remodeling was reduced in ischemic cardiomyopathy patients compared with non-ischemic cardiomyopathy patients; 2 compared with non-ischemic cardiomyopathy patients, ischemic cardiomyopathy patients exhibited more severe diastolic dysfunction; and 3 severe diastolic dysfunction (restrictive filling) was an independent factor influencing the CRT response.

Many randomized controlled trials and observational studies 3,4,13-15 have shown that CRT results in less improvement in LV systolic function and remodeling in ICM patients than in NICM patients. This study showed that the ICM group had lower CRT response rates six months after implantation; improvements in LVEF and LVOT-VTI, which reflect LV function, and improvements in LVESV, which reflect remodeling, were smaller in the ICM group than in the NICM group. These results are not surprising. Several groups reported similar findings as long as a decade ago 16-18. Hummel et al. proposed that their similar findings were associated with the amount of viable myocardium 19. Because the ICM group had a smaller amount of viable myocardium, the improvement in LV function due to CRT could not be maintained. Other studies have shown that myocardial scars are associated with CRT nonresponse 5,6,20, which has been described in relation to the myocardial scar burden, degree of transmurality, and locations of the scars and LV lead. Chalil et al. further proposed that a scar size ≥33%, transmurality ≥51%, and pacing over a posterolateral scar in ICM patients were associated with suboptimal CRT responses 7. However, the specific mechanism that causes different CRT curative effects in heart failure patients with different etiologies remains unclear. Waggoner et al. reported that even when ICM patients exhibited improved LV systolic function, their diastolic function was not improved compared with NICM patients, and this result was attributed to the presence of relatively restrictive LV filling before CRT in ICM patients and a lack of improvement of end-diastolic volume and diastolic synchrony after CRT 21. The present study also showed that the ICM group presented with elevated LV filling pressures (i.e., elevated mitral E/A and E/Em) before CRT and that LVEDV was not improved six months after CRT.

Although diastolic function is an important component of overall cardiac function, it has long received much less attention than systolic function. Abnormal diastolic function is important for the development of heart failure symptoms and signs. Even during systolic heart failure, increased LV filling pressure is closely associated with exercise limitation and is independent of the degree of systolic dysfunction 8. Studies have increasingly indicated that severe diastolic dysfunction (restrictive filling) suggests a worse hemodynamic and clinical status of heart failure as well as increased mortality and heart transplantation rates 22, which is also true for patients undergoing drug treatment 23,24, surgical ventricular restoration 25,26, and CRT 27,28. Using multivariate regression analysis, this study confirmed that severe diastolic dysfunction was an independent factor that influenced the CRT response.

Diastolic dysfunction is a typical presentation of ICM. Abnormal diastolic function occurs at the early stage of myocardial ischemia, even earlier than systolic dysfunction 29,30. During myocardial ischemia, ATP production, ATP-dependent Ca^2+^ pump activity, Ca^2+^ uptake into the sarcoplasmic reticulum, and Ca^2+^ outflow decrease, and myosin-actin complex dissociation disorder occurs, resulting in a delayed and incomplete active diastole 31. Long-term recurrent attacks of ischemia and infarction, myocardial necrosis, advanced interstitial fibrosis, scar formation, increases in ventricular stiffness, and reduction in compliance will cause passive diastolic dysfunction, sustained increase in ventricular filling pressure, and eventually, development of restrictive filling 32. This phenomenon is different from dilated cardiomyopathy, which mainly causes ventricular enlargement and systolic dysfunction. In this study, the ICM group exhibited more severe diastolic dysfunction than the NICM group. Therefore, we propose that in addition to a lack of viable myocardium and the presence of myocardial scars, the secondary severe diastolic dysfunction in ICM patients might also cause suboptimal improvement in LV function and structure after CRT.

### Limitations

This study is subject to the following limitations. 1 This was a single-center observational study with no randomized control group. 2 The composition of the underlying etiologies was not balanced; fewer cases of ICM were included. 3 Invasive measurements (such as left ventricle end diastolic pressure (LVEDP)) were not performed for the evaluation of diastolic function. Therefore, it is necessary to increase the sample size and validate invasive measurements to further clarify the reason for the different curative effects of CRT for different disease etiologies.

In summary, this study again confirmed that the etiology of heart failure affected the curative effects of CRT and that a lower degree of improvement of ventricular systolic function and remodeling was observed in ICM patients than in NICM patients. In addition, systolic heart failure patients with severe diastolic dysfunction showed poor CRT responses. ICM patients exhibited more severe diastolic dysfunction than NICM patients, which may be a reason for the reduced beneficial effect of CRT. The findings of our study are similar to those published as long as a decade ago, but the intervening period has seen little attention to this important aspect of the CRT response. We feel that it may now be reasonable to refocus on this aspect of CRT and that a study from an Asian country may reignite interest.

## AUTHOR CONTRIBUTIONS

Wang Q, Chen KY and Yan J conceived and designed the study. Wang Q and Chen KY drafted the manuscript. Wang Q, Hu Y and Yang DM were responsible for the acquisition of data. Chen KY, Yu F, An CS and Su H were responsible for the analysis and interpretation of data. Yan J was responsible for the administrative support. Xu J, Su H and Yang DM were responsible for the critical revision of the manuscript for important intellectual content. All authors have read and approved the final version of this manuscript.

## Figures and Tables

**Table 1:** Basic Patient Data.

Parameter	Total n=104	NICM n=77	ICM n=27	*p*-value (NICM vs. ICM)
Age, y	59.8±10.6	59.3±11.2	61.3±8.8	0.392
Male, n(%)	78 (75.0)	57 (74.0)	21 (77.8)	0.698
Disease course, y	5.2±2.9	5.3±3.0	5.0±2.7	0.600
NYHA class, n(%) III/IV	81 (77.9)/23 (22.1)	60 (77.9)/17 (22.1)	21 (77.8)/6 (22.2)	0.988
Heart rate (beats/min)	74.1±12.2	73.1±12.3	77.0±11.9	0.158
PR interval, ms	197.8±37.3	199.4±38.2	193.3±35.1	0.474
AV delay, ms	122.1±17.8	123.1±17.2	119.3±19.6	0.336
QRS duration, ms	154.3±24.8	155.6±24.7	150.7±25.0	0.384
SCr, μmol/l	88.8±29.5	88.3±28.5	90.1±32.7	0.794
LAD, mm	47.2±5.7	46.9±6.0	48.1±4.4	0.279
LVEDD, mm	74.8±8.0	74.7±7.6	75.2±9.1	0.771
LVESD, mm	64.8±7.8	64.4±7.4	65.7±9.0	0.454
LVEDV, ml	300.7±73.3	298.9±70.2	305.8±82.8	0.676
LVESV, ml	217.6±61.4	214.7±58.3	225.8±69.9	0.421
LVEF, %	28.2±4.9	28.4±4.7	27.4±5.4	0.332
FS, %	13.4±2.8	13.7±2.5	12.8±3.4	0.241
MR, n(%)				0.256
grade 1	22 (21.2)	17 (22.1)	5 (18.5)	
grade 2	68 (65.4)	52 (67.5)	16 (59.3)	
grade 3	14 (13.5)	8 (10.4)	6 (22.2)	
SPAP, mmHg	39.1±11.9	38.4±12.0	41.2±11.4	0.290
E, cm/s	75.9±25.6	71.7±25.8	87.8±21.3	0.005
A, cm/s	64.6±17.1	67.1±17.0	57.5±15.8	0.012
E/A	1.3±0.7	1.2±0.7	1.7±0.7	0.003
DT, ms	187.3±43.8	193.9±42.1	168.3±43.9	0.008
Em septal, cm/s	5.2±1.3	5.4±1.4	4.7±1.0	0.010
Em lateral, cm/s	7.8±1.7	7.9±1.6	7.5±2.0	0.253
E/Em	12.3±5.6	11.2±5.1	15.4±6.1	0.001
LVOT-VTI, cm	9.9±1.7	10.0±1.7	9.6±1.5	0.242
Ts-SD, ms	70.3±31.5	72.6±30.6	63.8±33.7	0.215
RFP, n(%)	33 (31.7)	19 (24.7)	14 (51.9)	0.009
Diuretics, n(%)	104 (100)	77 (100)	27 (100)	1.000
ACE-I or ARB, n(%)	86 (82.7)	66 (85.7)	20 (74.1)	0.280
β-Blocker, n(%)	73 (70.2)	54 (70.1)	19 (70.4)	0.981
Amiodarone, n(%)	51 (49.0)	34 (44.2)	17 (63.0)	0.093
Spironolactone, n(%)	100 (96.2)	75 (97.4)	25 (92.6)	0.591

SCr, serum creatinine; LVEDV, left ventricular end diastolic volume; LVESV, left ventricular end systolic volume; LVEF, left ventricular ejection fraction; MR, mitral regurgitation; SPAP, systolic pulmonary arterial pressure; LVOT-VTI, left ventricular outflow tract-velocity time integral; ACE-I, angiotensin-converting enzyme inhibitors; ARB, angiotensin receptor blockers.

**Table 2:** Clinical and echocardiographic characteristics of the overall population at baseline and follow-up.

Parameter	Baseline	Follow-up	*p*-value
NYHA class, I/II/III/IV	0/0/81/23	15/58/23/8	<0.001
LAD, mm	47.2±5.7	45.9±6.5	<0.001
LVEDV, ml	300.7±73.3	279.8±84.9	<0.001
LVESV, ml	217.6±61.4	183.0±72.9	<0.001
LVEF, %	28.2±4.9	36.0±8.9	<0.001
FS, %	13.4±2.8	17.6±4.7	<0.001
SPAP, mmHg	39.1±11.9	37.8±12.8	NS
MR grade, 1/2/3	22/68/14	42/43/19	0.043
LVOT-VTI, cm	9.9±1.7	12.9±3.8	<0.001

**Table 3:** Clinical and echocardiographic characteristics of patients with ICM and NICM.

Parameter	NICM	ICM	*p-*value
	n=77	n=27	
NYHA class, I/II/III/IV			
Baseline	0/0/60/17	0/0/21/6	NS
Follow-up	12/45/16/4*	3/13/7/4#	NS
LAD, mm			
Baseline	46.9±6.0	48.1±4.4	NS
Follow-up	45.5±6.6*	47.0±6.2	NS
LVEDV, ml			
Baseline	298.9±70.2	305.8±82.8	NS
Follow-up	272.6±79.9*	300.4±96.5	NS
LVESV, ml			
Baseline	214.7±58.3	225.8±69.9	NS
Follow-up	174.5±67.2*	207.2±84.0#	0.045
LVEF, %			
Baseline	28.4±4.7	27.4±5.4	NS
Follow-up	37.3±8.4*	32.4±9.3#	0.013
FS, %			
Baseline	13.7±2.5	12.8±3.4	NS
Follow-up	18.3±4.3*	15.6±5.2#	0.008
SPAP, mmHg			
Baseline	38.4±12.0	41.2±11.4	NS
Follow-up	36.0±12.1#	42.9±13.7	0.015
MR grade, 1/2/3			
Baseline	17/52/8	5/16/6	NS
Follow-up	33/34/10#	9/9/9	NS
LVOT-VTI, cm			
Baseline	10.0±1.7	9.6±1.5	NS
Follow-up	13.5±3.5*	11.1±3.9#	0.003

* *p*<0.001;

# *p*<0.05; follow-up vs. baseline.

**Table 4:** Univariate and multivariate logistic regression analyses: estimates of correlations between baseline clinical and echocardiographic characteristics and response to CRT.

Parameter	Univariate		Multivariate	
	HR (95% CI)	*p*-value	HR (95% CI)	*p*-value
Age, y	0.986 (0.949-1.025)	0.487		
Male sex	0.261 (0.082-0.831)	0.023		
Ischemic etiology	0.341 (0.138-0.840)	0.019		
Disease course, y	0.815 (0.701-0.948)	0.008		
NYHA class IV	0.487 (0.189-1.252)	0.135		
SCr, μmol/l	0.979 (0.963-0.994)	0.007		
QRS duration, ms	1.038 (1.016-1.060)	0.001	1.045 (1.013-1.077)	0.006
LAD, mm	0.821 (0.749-0.901)	<0.001	0.846 (0.730-0.980)	0.026
LVEDV baseline, ml	0.997 (0.992-1.003)	0.297		
LVEF baseline, %	1.019 (0.938-1.108)	0.653		
SPAP baseline, mmHg	0.919 (0.879-0.961)	<0.001		
MR baseline grade 3	0.221 (0.052-0.944)	0.042		
Ts-SD, ms	1.006 (0.993-1.020)	0.361		
RFP	0.123 (0.048-0.311)	<0.001	0.100 (0.025-0.397)	0.001

HR, indicates hazard ratio; CI, confidence interval.
